# Herpes-Virus Infection in Patients with Langerhans Cell Histiocytosis: A Case-Controlled Sero-Epidemiological Study, and In Situ Analysis

**DOI:** 10.1371/journal.pone.0003262

**Published:** 2008-09-23

**Authors:** Eric Jeziorski, Brigitte Senechal, Thierry Jo Molina, Francis Devez, Marianne Leruez-Ville, Patrice Morand, Christophe Glorion, Ludovic Mansuy, Joel Gaudelus, Marianne Debre, Francis Jaubert, Jean-Marie Seigneurin, Caroline Thomas, Irene Joab, Jean Donadieu, Frederic Geissmann

**Affiliations:** 1 Laboratory of biology of the mononuclear phagocyte system, INSERM U838, University Paris-Descartes, Paris, France; 2 Hopital de l'Hotel Dieu, Pathology department, AP-HP, Paris, France; 3 Hopital Necker-Enfants Malades, AP-HP, Paris, France; 4 Centre Hospitalo-Universitaire Michallon, Virology department, Grenoble, France; 5 Centre Hospitalo-Universitaire de Nancy, Medecine infantile II, Nancy, France; 6 Hopital Jean Verdier, AP-HP, service de Pediatrie, Bondy, France; 7 Centre Hospitalo-Universitaire de Nantes, Pediatrie, Nantes, France; 8 UMR542 Inserm-Universite Paris Sud, Hopital Paul Brousse, Villejuif, France; 9 Hopital d'Enfants Armand Trousseau, Pediatric Hematology unit, Centre de référence de l'histiocytose AP-HP, Paris, France; Karolinska Institutet, Sweden

## Abstract

**Background:**

Langerhans cell histiocytosis (LCH) is a rare disease that affects mainly young children, and which features granulomas containing Langerhans-type dendritic cells. The role of several human herpesviruses (HHV) in the pathogenesis of LCH was suggested by numerous reports but remains debated. Epstein-barr virus (EBV, HHV-4), & Cytomegalovirus (CMV, HHV-5) can infect Langerhans cells, and EBV, CMV and HHV-6 have been proposed to be associated with LCH based on the detection of these viruses in clinical samples.

**Methodology:**

We have investigated the prevalence of EBV, CMV and HHV-6 infection, the characters of antibody response and the plasma viral load in a cohort of 83 patients and 236 age-matched controls, and the presence and cellular localization of the viruses in LCH tissue samples from 19 patients.

**Principal Findings:**

The results show that prevalence, serological titers, and viral load for EBV, CMV and HHV-6 did not differ between patients and controls. EBV was found by PCR in tumoral sample from 3/19 patients, however, EBV small RNAs EBERs –when positive-, were detected by in situ double staining in bystander B CD20^+^ CD79a^+^ lymphocytes and not in CD1a^+^ LC. HHV-6 genome was detected in the biopsies of 5/19 patients with low copy number and viral Ag could not be detected in biopsies. CMV was not detected by PCR in this series.

**Conclusions/Significance:**

Therefore, our findings do not support the hypothesis of a role of EBV, CMV, or HHV-6 in the pathogenesis of LCH, and indicate that the frequent detection of Epstein-barr virus (EBV) in Langerhans cell histiocytosis is accounted for by the infection of bystander B lymphocytes in LCH granuloma. The latter observation can be attributed to the immunosuppressive micro environment found in LCH granuloma.

## Introduction

Langerhans cell histiocytosis (LCH, a.k.a. histiocytosis X), is a rare disease that affects mainly young children, and features granulomas consisting of Langerhans-like cells (LC), mixed with macrophages, eosinophiles, multinucleated giant cells, and lymphocytes, that can be found within various tissues [1], [2].

The presence of LC in granuloma is a key diagnostic feature of LCH. LC are members of the dendritic cell (DC) family, that trigger and shape immune responses, and the pathophysiology of LCH is likely to involve immune mechanisms (reviewed in [3], [4]). We have previously reported that LC found in LCH granuloma were phenotypically and functionally immature/semi-mature LC [5]. Immature/semi-mature DCs are believed to be prone to induce regulatory T cells, that inhibit polyclonal T cell responses and promote tolerance [6], [7], [8], [9]. The accumulation of immature LC in LCH granulomas was associated with the expansion of FoxP3^+^ CD25 + CD4^+^ regulatory T cells both in granuloma and in the blood of patients [10]. Therefore local and general immunosuppression, which favors reactivation of herpes-virus infection, may be a feature of LCH.

Environmental agents and viruses, in particular Epstein-Barr virus (EBV), or vaccination, have been proposed to trigger, or to play a role in the pathogenesis of the disease [11], [12]. Herpesviruses are DNA viruses responsible for persistent infection. EBV is the etiological agent of several malignancies [13], [14], and EBV & Cytomegalovirus (CMV) are responsible for hemophagocytic syndromes in human with several inherited immunodeficiencies [15], [16]. EBV has been reported to infect monocytes and Langerhans cells (LC), during the natural course of infection in human [17], [18]. Infection with EBV has been reported to be associated with LCH, to represent a possible etiology, and/or to contribute to its pathophysiology in some studies [11], [12], [19]. However, other studies failed to replicate these findings, and the possible causative role of EBV in LCH is debated [20], [21], [22]. CMV can also infect DC and LC [23], [24], [25], and one single study reported CMV detection in lesional LC in one third of 29 patients by immunohistochemistry, in situ hybridization, and PCR [26]. HHV-6 infects mainly T cells, but is also reported to infect myeloid cells [27], [28] and HHV-6 DNA or immunoreactivity was detected in lesions of 50% to 75% of patient with LCH [29], [30], however, control studies performed by the same group concluded that the prevalence of HHV-6 in the tissue of LCH patients is the same as that found in tissue from individuals without disease [31].

Sero-epidemiological studies have been useful to demonstrate the role of EBV in Burkitt lymphoma and Hodgkin disease, when high antibody titers to EBV structural antigens (VCA) have been associated to the risk of developing Burkitt's lymphoma and Hodgkin diseases [32], [33]. However, to our knowledge no sero-epidemiological study have been conducted in LCH. The present study was therefore designed to investigate the role of EBV, CMV, and HHV-6 using two methods. First, we performed a case-controlled sero-epidemiological study to investigate a relationship between the onset of LCH in young children and the antibody response to infection with EBV, CMV, or HHV-6, and second we searched for the presence of viruses in the serum of patients, and in Langerhans cells in tumor samples, by PCR and, when positive, we investigated the cellular target of the viruses by immunolabeling and in situ hybridization. Results ruled out an epidemiological association between these herpes-virus and LCH.

## Results

Prevalence of EBV, CMV, and HHV-6 infection as a function of age and of clinical presentation of disease. The presence of antibodies against EBV, CMV and HHV-6 was investigated by ELISA in the serum of 78 children diagnosed with LCH and 206 age matched controls. To avoid contamination by maternal IgG, only children six month-old and older where studied for the presence of specific antibodies in serum. Twenty six children with LCH (33.3%) and 94 controls (47.1%) tested seropositive for EBV. No significant difference was observed between patient and control groups when matched for age (Table 1). Similarly, no significant difference was observed with the control group when patients were grouped by disease stage/clinical presentation (Table 2). Similar results were observed for CMV and HHV-6, and the prevalence of both infections in children with LCH were comparable to their prevalence among age-matched controls (Tables 2,and 3).

**Table 1:** Prevalence of EBV, CMV, and HHV-6 infection in LCH patients

		Controls	LCH total	LCH1	LCH2	LCH3
EBV						
Non-infected	n	109	52	28	12	12
	%	52.9	66.7	65.1	63.2	75
Infected	n	97	26	15	7	4
	%	41.1	33.3	34.9	36.8	25
Total	n	206	78	43	19	16
	%	100	100	100	100	100
Test Fisher's exact	0.475					
CMV						
Non-infected	n	131	58	32	13	13
	%	64.2	75.3	76.2	68.4	81.2
Infected	n	72	18	9	6	3
	%	35.3	23.4	21.4	31.6	18.8
Unknown*	n	1	1	1	0	0
	%	0.5	1.3	2.4	0	0
Total	n	204	77	42	19	16
	%	100	100	100	100	100
Test Fisher's exact	0.282					
HHV-6						
Non-infected	n	33	15	5	5	5
	%	1.4	19.5	11.9	26.3	31.2
Infected	n	161	62	37	14	11
	%	80.1	80.5	88.1	7.7	68.7
Unknown*	n	7	0	0	0	0
	%	3.5	0	0	0	0
Total	n	201	77	42	19	16
	%	100	100	100	100	100
Test Fisher's exact	0.424					

Unknown^*^: IgG value are in the ‘grey zone’ (see methods)

LCH1: single system, no risk organs

LCH2: multisystem, no risk organs

LCH3: risk organs

**Table 2:** Prevalence of EBV, CMV, and HHV-6 infection as a function of age

		Age 0.5 to 5		Age 5 to 10		Age 10 to 20	
		Controls	LCH	Controls	LCH	Controls	LCH
EBV							
Non-infected	n	79	35	19	11	11	6
	%	71.8	85.4	35.8	45.8	25.6	46.1
Infected	n	31	6	34	13	32	7
	%	28.2	14.6	64.1	54.2	74.4	53.8
Total	n	110	41	53	24	43	13
	%	100	100	100	100	100	100
Test Fisher's exact			0.093		0.577		0.378
CMV							
Non-infected	n	74	34	29	13	27	11
	%	69.4	85	54.7	54.2	62.8	84.6
Infected	n	33	6	23	10	16	2
	%	30.6	15	43.4	41.7	37.2	15.4
Unknown*	n	0	0	1	1	0	0
	%	0	0	1.9	4.2	0	0
Total	n	107	40	53	24	43	13
	%	100	100	100	100	100	100
Test Fisher's exact			0.062		0.82		0.186
HHV-6							
Non-infected	n	18	14	5	0	10	1
	%	17	35	9.4	0	23.8	7.7
Infected	n	84	26	47	24	30	12
	%	79.2	65	88.7	100	71.4	92.3
Unknown*	n	4	0	1	0	2	0
	%	3.8	0	1.9	0	4.8	0
Total	n	106	40	53	24	42	13
	%	100	100	100	100	100	100
Test Fisher's exact			0.037		0.217		0.356

**Table 3:** Prevalence of HHV-6 infection in children before the age of 5 years

		Age 0.5 to 1		Age 1 to 1.5		Age 1.5 to 2		Age 2 to 5	
		controls	LCH	controls	LCH	controls	LCH	controls	LCH
Non-infected	n	5	8	0	2	4	0	9	4
	%	35.7	66.7	0	33.3	30.8	0	14.8	21
Infected	n	9	4	17	4	9	3	49	15
	%	64.3	33.3	95.4	66.7	69.2	100	80.3	79
Unknown*	n	0	0	1	0	0	0	3	0
	%	0	0	5.6	0	0	0	4.9	0
Total	n	14	12	18	6	13	3	61	19
	%	100	100	100	100	100	100	100	100
Test Fisher's exact			0.238		0.054		0.529		0.663

**Figure 1:**
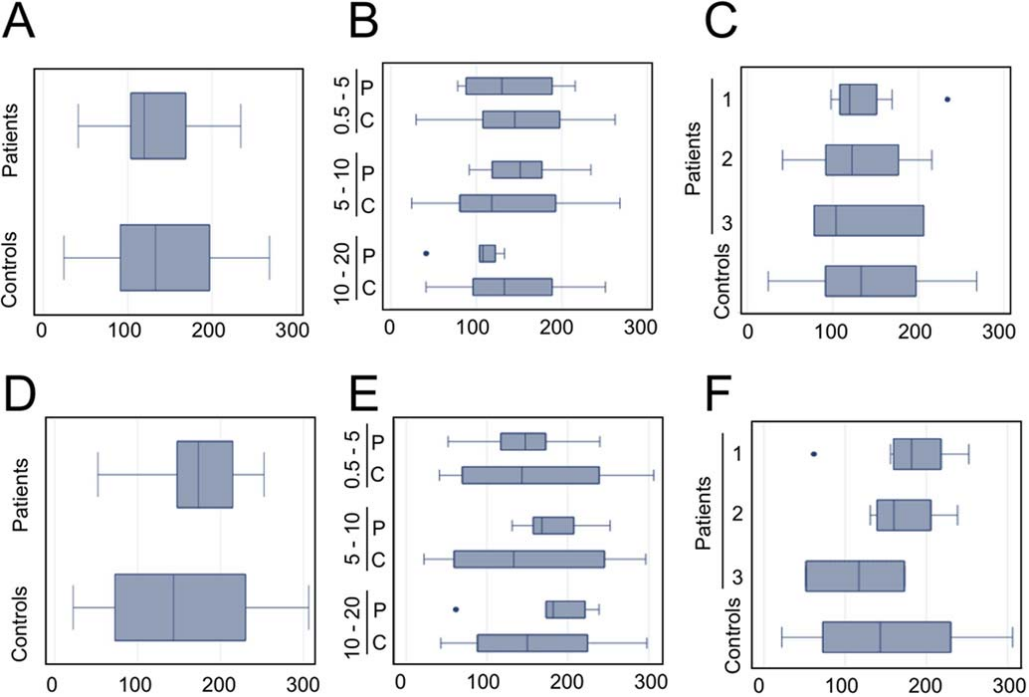
VCA-IgG and EBNA-IgG titers (UA/ml) in patients and controls with past EBV infection. VCA-IgG (A, B, C) and EBNA-IgG titers (D, E, F) (UA/ml) were determined as indicated in methods, and patients were compared to controls without stratification (A, D), and after stratification based on age (B, E), or disease extension (C, F).

Serum IgG titers directed against EBV, as well as CMV and HHV-6, did not differ between patients and controls. Titers of EBV antibodies have been shown to differ from controls in several diseases linked to EBV [14], [34]. IgG VCA titers, and in some cases anti-Epstein-Barr nuclear antigen (EBNA)1 antibody, are consistently higher in patients with nasopharynx carcinoma or Hodgkin disease than in control populations [14]. Lower EBNA1-IgG antibody titer is also considered as a possible serological sign for a defective control of the persistent latent EBV carrier state. In the present study, both VCA IgG and EBNA IgG titers were found to be similar in patients with LCH in comparison with controls, when matched for age, or when grouped by disease stage/clinical presentation (Figure 1A–E). Anti HHV-6 or anti CMV IgG titers were also not different between patients and the control group (data not shown)

Prevalence of detectable serum viral load for EBV, CMV, and HHV-6. Defective control of infection by Herpesviruses such as EBV, CMV and HHV-6 ultimately results in replicative infection and viremia [35]. Serum viral load during CMV infection is a sensitive technique, but in the present study, out of 83 patients and 236 age-matched controls, 0% of patient and 3% of controls (n = 7) had detectable CMV serum viral load (table 4) indicating the very low indidence of active infection. Serum viral load during EBV infection is considered to be less sensitive than during CMV infection [36], nevertheless we found that only 1.2% of patients (n = 1) and a similar percentage (0.9% of controls, n = 2) had a detectable EBV viral load in serum (Table 4). These results are consistent with anti-VCA IgG titers, which were found similar in patients and controls (see figure 1), since EBV viral load was shown to be correlated with anti-VCA IgG titer [34]. The significance of the detection of HHV-6 in the serum is still discussed [37], [38], but again only 3.6% of patients (n = 3) and 2.5% of controls (n = 6) had a detectable HHV-6 serum viral load, and there was no difference between groups as per fischer exact's test (Table 4).

**Table 4:** Prevalence of detectable serum viral load for EBV, CMV, and HHV-6

	Patients	Controls	Total
EBV			
Negative	82	234	316
%	98.8	99.1	99.1
Positive	1	2	3
%	1.2	0.9	0.9
Total	83	236	319
%	100	100	100
Fisher's exact = 1.000			
1-sided Fisher's exact = 0.596			
CMV			
Negative	83	229	312
%	100	97.	97.8
Positive	0	7	7
%	0	3	2.2
Total	83	236	319
%	100	100	100
Fisher's exact = 0.197			
1-sided Fisher's exact = 0.118			
HHV-6			
Negative	80	230	310
%	96.4	97.5	97.2
Positive	3	6	9
%	3.6	2.5	2.8
Total	83	236	319
%	100	100	100
Fisher's exact = 0.701			
1-sided Fisher's exact = 0.428			

Detection of EBV, CMV, and HHV-6 DNA in biopsy samples from LCH granuloma. We then investigated whether Herpesviruses were present in LCH granuloma, in the absence of overt viremia. As shown in table 5, EBV DNA was found in 15% of biopsy samples examined (3/19 patients, #1, 9, & 15), and HHV-6 DNA in 26% of biopsy samples examined (5/19 patients). CMV DNA was not detected in this series. EBV and HHV-6 viral loads were low with a median of 400 copies/10^6^ cells except for patient #5. Patient #5 display a very high HHV-6 load in the biopsy and in the blood over time (data not shown) compatible with the detection of a chromosomally integrated HHV-6 DNA [39].

**Table 5:** Detection of EBV, CMV, and HHV-6 DNA in biopsy samples from LCH granuloma.

Patient	stage	sex	HHV-6	EBV	CMV
1	LCH1	F	-	6 10^3^	-
2	LCH1	M	8 10^2^	-	-
3	LCH1	M	-	-	-
4	LCH1	F	6 10^1^	-	-
5	LCH1	M	2 10^6^	-	-
6	LCH1	F	-	-	-
7	LCH1	M	-	-	-
8	LCH1	F	-	-	-
9	LCH1	F	-	1.7 10^5^	-
10	LCH1	F	-	-	-
11	LCH1	M	-	-	-
12	LCH1	M	-	-	-
13	LCH1	M	-	-	-
14	LCH1	F	-	-	-
15	LCH1	M	-	4 10^2^	-
16	LCH2	M	4 10^2^	-	-
17	LCH3	M	-	-	-
18	LCH3	M	-	-	-
19	LCH3	M	4 10^2^	-	-

Positive results are expressed as the number of viral genome copies per million cells.

Therefore, our data support previous results showing the presence of EBV or HHV-6 in a subset of LCH granuloma. However, since HHV-6 and EBV are responsible for persistent infection of T cells and B cells respectively, the detection of virus DNA in LCH samples could be attributed to infection of bystander cells [21].

**Figure 2:**
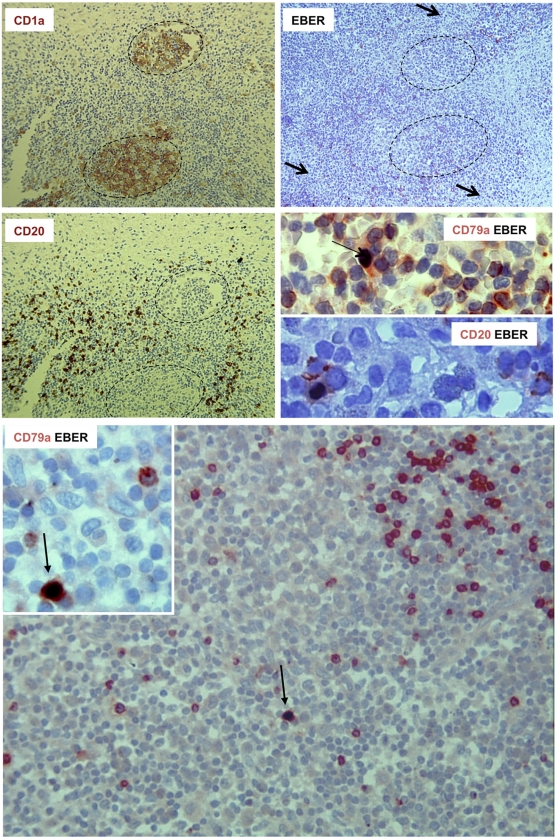
Detection of EBV-infected B cells by in situ hybridization combined with immunohistochemistry. Granuloma serial sections were stained for CD1a (upper left) or for CD20 (middle left) by immunohistochemistry or for EBERs by in situ hybridisation (upper right, arrows indicate EBER positive cells). Combined detection of CD20 or CD79a with EBERs shows that EBV-infected cells are B cells (arrows).

We therefore investigated whether EBV infects bystanders lymphocytes, macrophages, and Langerhans cells in samples from patients #1, 9, & 15, which tested positive by PCR, using in situ hybridisation with a probe against EBV EBERs RNA on paraffin sections of LCH granuloma, followed by immunohistochemistry with antibodies against B-cells (CD20 & CD79a), T-cells (CD3), macrophages (CD68), or Langerhans cell (CD1a) antigens on the same tissue sections. EBV positive cells were always found in areas rich in B cells, and were labeled with either CD20, or CD79a antibodies (Figure 2), while no EBER+ cell labelled with CD1a, CD3, or CD68 cell was observed. These data indicate that EBV infects bystander B cells, which are present in a subsets of LCH granuloma [12]. Although positive controls were obtained, we did not observed immunoreactivity against HHV-6 in samples positive for HHV-6 DNA by PCR (data not shown), therefore the cellular target of HHV6 in LCH granuloma was not identified.

## Discussion

The present study is the largest case-controlled sero-epidemiological study performed in this disease. The results argue against an epidemiological association between LCH and EBV, CMV, or HHV-6 infection.

Further investigation of the cellular localization of the viruses in LCH tissue samples from 19 patients indicated that, when present, EBV infected bystander lymphocytes and not Langerhans cells. We think that our results therefore resolve the much debated issue of the role of EBV in the pathogenesis of LCH [19], [21], [22], [30], [31]. Because EBV infected B cells are present in the blood of healthy seropositive individuals [40] at a low frequency in the order of 10-^6^ [41] it is not unexpected to find a small number of EBV infected B cells in LCH granuloma, which contains B cells. In addition, because the growth of EBV infected B cells is under the control of CD8 cytotoxic T cells in healthy individuals [42], the regulatory T cell-rich environment of the LCH granuloma [10], may represent a sanctuary for such EBV infected B cells.

Anti CMV IgG titers in patients and controls, and absence of detection of the virus by PCR in the serum and in biopsy samples strongly suggest that CMV is not associated with LCH.

The case of HHV6 is more complicated. HHV-6 was detected by PCR in the serum of 3.8% of patients and 2.5% of controls with viral loads above 10^4^ copies/ml in 5 out of 9 detected (data not shown). The prevalence of HHV-6 in patients and controls were similar to the reported prevalence of chromosomally integrated HHV6 among blood donors [39]. Therefore HHV6 detection may be related to the presence of chromosomally integrated HHV-6. HHV6 DNA was also detected by PCR in biopsies from 5/19 patients (26%), suggesting that HHV6, like EBV can be found in LCH granuloma. However the viral load was very low, and the virus was not detectable by immunochemistry suggesting that HHV-6 infection is quiescent. Together with our serological data and serum PCR, and in the light of the study of Glotzbecker et al, [31] who found a similar frequency PCR positive samples in LCH granuloma and control tissues, we hypothetize that HHV6 do not productivey infect LC in vivo and may be carried by bystander lymphocytes which are present in the granuloma.

Our study did not identify a subset of patients with ‘herpes-viruses associated’ LCH. We did not found an association between EBV, CMV, or HHV-6 infection and patients when they were stratified by age, or by clinical stage.

Among other herpes viruses, HHV-1 (HSV-1), HHV-2 (HSV-2), HHV-3 (VZV) and HHV-7 infections were never reported to be associated with LCH, and were not investigated in this study. Among herpesviruses, HHV-8 has been initially reported to infect dendritic cells, but several studies have excluded its association with LCH [43], [44].

## Methods

### Patients & controls

The diagnostic and inclusion criteria as well as the definition of the organs involvement for the French nationwide LCH survey have been described elsewhere [2]. Briefly, the extension of the diseases has been classified in three groups according to histiocyte society criteria: group 1: single organ extension, without risk organs group 2 mulsystem organ without risk organs and group 3 patients with risk organs i.e. lung and/or liver and/or spleen and/or hematological dysfunction. According to French bioethics laws, informed consent was signed if the patients participated and the database was approved by the French computer watchdog commission (CNIL certificate n° 99.087). Clinical information, radiological findings and extension were recorded, together with treatments received. Data monitoring, based on medical charts, was done by a clinical research associate who visited each center. Involvement of at least one new organ, as described elsewhere 2, was considered to define an LCH episode. Serum specimens were obtained, after written witnessed informed consent was obtained from the parents of all patients, from 83 pediatric patients with LCH included in the French LCH registry, following a research protocol approved by the ethics committee of the Nantes University Hospital (France, EU). Biopsies samples were also obtained from 19 patients with LCH included in the French LCH registry after written witnessed informed consent was obtained. Control serum samples were obtained from the children admitted to the outpatient unit of the Grenoble University Hospital (France, EU), according to institutional guidelines, and were matched for age with patient samples for statistical analysis.

### Serology

Qualitative and quantitative analysis of specific Ig against EBV, CMV and HHV-6 were performed on serum samples from 83 patients and 235 controls using microplate ELISA kits: IgG anti-VCA (ETI-VCA-G, DiaSorin®), IgG anti-EBNA (ETI-EBNA-G, DiaSorin®), IgM anti-VCA (DiaSorin®,ETI-EBV-M), IgG anti-CMV(ETI-CYTOK-G PLUS, DiaSorin®) and IgG anti-HHV-6 (HHV-6 IgG EIA, Biotrin®). Patients and controls were classified in three categories according to the detection of specific IgG i/above the upper limit of the grey zone for “infected” status, ii/in the grey zone for “unknown” status, iii/below the lower limit of the grey zone for “uninfected” status.

### DNA extraction from frozen biopsies and serum

DNA was extracted from sliced frozen biopsies using ALLPrep DNA/RNA minikit (Qiagen, Hilden, Germany). Total DNA was eluted with 200 ul of water and amplification of the beta-globin gene by real time PCR was used to evaluate total cell number per sample (Beta-globine PCR Kit, Roche Diagnostics®). DNA was extracted from 200ul of serum using Blood DNA mini kit and eluted in a volume of 100ul.

### Quantitative PCR for the detection of EBV, CMV and HHV-6

ten ul of DNA extract were used to detect and quantify viral genomes by real time PCR assays. All positive PCR were run a second time for confirmation.

EBV tyrosine kinase gene (TK) was amplified from biopsies DNA with T1 (5′-GGGGCAAAATACTGTGTTAG-3′)+T2 (5′-CGGGGGACACCATAGT-3′) primers and LC1 (5′-ATGTTTCCTCCCTCGCTTCTTCAG-fluo-3′)+LC2 (5′-ATGTTTCCTCCCTCGCTTCTTCAG-fluo-3′) probes. PCR were run on a Light Cycler. The EBV-negative DG75 cell line was used as a negative control. The EBV-positive Burkitt's lymphoma cell line “Namalwa” [45] harboring 2 copies of viral genome per cell is used as a standard for quantification. Detection limit is 2 copies of viral genome per amplification [46].

CMV immediate early-1 gene (IE1) was amplified using forward 5′-GCAGACTCTCAGAGGAT-3′+reverse 5′-AGCGCCGCATTGAGGA-3′ primers and a 6-carboxyfluoresceine (FAM) -5′ ATCTGCATGAAGGTCTTTGCCCAGTACATT-3′ carboxytetramethyl rhodamine (TAMRA) probe. PCR were run on a ABI 7300 real time PCR system (Applied Biosystem).Quantification was obtained using a plasmid and the detection limit is 20 copies of viral genome per amplification [35].

HHV-6 U65-U66 gene was amplified using forward 5′- GACAATCACATGCCTGGATAATG-3′+reverse 5′-TGTAAGCGTGTGGTAATGGACTAA -3′ primers and a 6-carboxyfluoresceine (FAM) -5′ AGCAGCTGGCGAAAAGTGCTGTGC-3′ carboxytetramethyl rhodamine (TAMRA) probe. PCR were run on a ABI 7300 real time PCR system (Applied Biosystem).Quantification was obtained using a plasmid and detection limit is 25 copies of viral genome per amplification [47].

PCR results were expressed as the number of viral genome copies per million cells for biopsies and as the number of viral genome copies per ml of serum from peripheral blood.

### In situ hybridization & immunohistochemistry

Detection of EBV EBERs RNA by in situ hybridization was performed first and was followed by immunohistochemistry with antibodies against B-cell, T-cell and Langerhans cell antigens. Five micrometers paraffin-embedded sections were mounted onto glass slides and pretreated in 0.4% pepsine HCl 0.2M, then hybridized with EBER PNA probes (DAKO), following the manufacturer's instructions, and revealed with NBT/BCIP after APAAP amplification. Immunohistochemistry was then performed using a streptavidin-biotin peroxidase method (LSAB2, DAKO) after microwave heating, revealed with AEC or DAB. Antibodies used were directed against the following antigens : CD20 (L26, 1∶200, Dako,), CD79a (JCB117, 1∶50, Dako), CD3 (UCHT1, 1∶50 Dako), CD1a (MTB1, 1∶25,Novocastra), CD68 (KP1, 1∶200, Dako).

### Statistical methods

Stata Software® version 8 was used for all statistical analyses. Categorical data were compared by using Fisher's exact test, and quantitative data (titer if seropositivity) by using Kruskal-Wallis non parametric test. All tests were two-tailed. P values of less than 0.05 were considered to indicate statistical significance unless otherwise stated.
